# Microrna-206 induces hypoxic necrosis of femoral head by inhibiting VEGF/PI3K/AKT signaling pathway

**DOI:** 10.3389/fgene.2023.1118831

**Published:** 2023-02-22

**Authors:** Xingjing Wu, Zhoushan Tao, Wenjing Cheng

**Affiliations:** ^1^ Department of Orthopedics, Yijishan Hospital, Wannan Medical College, Wuhu, Anhui, China

**Keywords:** hypoxic necrosis of femoral head, VEGF/PI3K/Akt signal pathway, microRNA-206 molecule, chemotherapy drug delivery system, anoxic necrosis

## Abstract

The most common form of non-traumatic necrosis of the femoral head is anoxic necrosis of the femoral head, which is a metabolic disease, mainly involving young and middle-aged people. Apoptosis and its related signal regulation pathway play an important role in the occurrence and development of hypoxic necrosis of the femoral head. In order to investigate the possible pathological manifestations of miR-206 and VEGF/PI3K/AKT signal pathway genes and their interactions in hypoxic necrosis of the femoral head, this paper intended to systematically study the expression and regulation mechanism of miR-206 and VEGF/PI3K/AKT signal pathway genes. The interaction between miR-206 and VEGF/PI3K/AKT signaling pathway and its regulation on apoptosis, differentiation and proliferation of human osteoblast cell line hFOB1.19 (SV40 transfer of human osteoblasts) were studied by double luciferase reporter gene analysis, overexpression and inhibition of miR-206, and gene silencing of VEGF/PI3K/AKT signaling pathway. After 24 h and 48 h of intervention with MicroRNA 206 on osteoblasts, it was found that the fluorescence intensity of caspase-3 was higher than that of 0 h group (*p* < 0.05). This paper has provided an important research basis for the research of femoral head necrosis and the development of new diagnosis and therapeutic drugs for this kind of disease. It also has provided a reference for the further promotion of the chemotherapy drug delivery system.

## 1 Introduction

MiR-206 is involved in the differentiation of osteoblasts and its expression level is significantly increased in the tissue of induced hypoxic necrosis of the femoral head. However, its specific role and molecular mechanism in the process of steroid induced necrosis of the femoral head remain unknown. Non-traumatic osteonecrosis of the femoral head (NONFH) is the most common orthopedic disease in clinic, most of which are accompanied by a history of corticosteroid use or a long history of alcoholism. The necrosis of femoral head is mostly due to the death of osteoblasts and bone marrow components caused by blood stasis of femoral head vein, damage or interruption of arterial blood supply, which leads to the change and collapse of femoral structure. Pain in hips, buttocks and groins is the main clinical pain, which often occurs in people aged 20–59 years. Due to the impact of COVID-19 in recent years, the clinical use of hormone drugs may further lead to an rise in the proportion of patients, which have a great impact on the quality of life, economic burden and social health undertakings. As a common difficult and miscellaneous disease in the field of orthopedics, NONFH has attracted extensive attention from researchers in other countries. This study of the functional texture of MicroRNA-206 has set a good precedent for the introduction of chemotherapy drug delivery system. The mechanism of MicroRNA-206 inducing hypoxic necrosis of the femoral head by inhibiting VEGF/PI3K/AKT signal pathway has been explored, which is helpful to understand more treatment methods for necrosis of the femoral head.

Femoral head necrosis is seriously affecting people’s physical and mental health. Sheng Zhai believed that hormone-induced necrosis of the femoral head is caused by long-term use of hormones (Sheng et al., 2021). Hua Kun-chi believed that core decompression was an important method to treat femoral head necrosis (Hua et al., 2019). Li Wei believed that in osteonecrosis of the femoral head (ONFH), the blood supply was insufficient to meet the metabolic needs of bone (Li et al., 2017). Zhang Ying believed that bone microcirculation might lead to bone necrosis related to ischemia and hypoxia (Zhang Y. et al., 2017). Leibold Christiane Sylvia performed hip dislocation and varus osteotomy on nine patients with femoral head necrosis (Leibold et al., 2020). However, the treatment of femoral head necrosis proposed by them lacks preciseness, so this paper introduced MicroRNA 206 for comparative optimization.

MicroRNA-206 can enhance cell toxicity. Luo pan believed that the incidence rate of steroid induced ONFH in patients receiving long-term treatment was 9%–40% (Luo et al., 2018). Ma Jian-xiong believed that the mechanism of ONFH was still unclear (Ma et al., 2017). Narayanan Aswath suggested surgical treatment for patients with advanced disease of avascular necrosis of femoral head (Narayanan et al., 2017). Landgraeber Stefan believed that advanced core decompression (ACD) was a new technology for the treatment of ONFH (Landgraeber et al., 2017). Shu Peng believed that ONFH was a common disabling joint disease (Shu et al., 2020). MicroRNA-206 was not combined with femoral head necrosis.

The tumor suppressor PDCD4 (programmed cell death4) plays an significant role in regulating the apoptosis of cardiovascular cells, and the expression of PDCD4 gene is regulated by multiple microRNAs. However, its pathological role and molecular mechanism in the process of human steroid induced avascular necrosis of the femoral head are still unclear. In order to investigate the potential pathological function and interaction of miR-206 and PDCD4 genes in steroid induced avascular necrosis of the femoral head, the functions and mechanisms of miR-206 and PDCD4 expression and their interaction in osteoblast apoptosis and steroid induced avascular necrosis of the femoral head are systematically studied. The correlation coefficient (R value) between miR-206 and mRNA level of VEGF/PI3K/AKT signaling pathway is about 0.48.

## 2 Method of inducing hypoxic necrosis of femoral head with microRNA-206 depending on chemotherapy drug delivery system

### 2.1 Mechanism of hypoxic necrosis of femoral head

Modern medicine of avascular necrosis of femoral head is based on precise anatomy and pathology. According to its clinical manifestations, this disease can be attributed to the category of bone arthralgia, bone erosion and hip arthralgia in traditional Chinese medicine. Hip joint is the main mechanical conversion mechanism and key load-bearing part of the human body. It is mainly composed of acetabulum and femoral head. The pathogenesis of the disease is complex. It is widely believed that alcohol, hormone, pregnancy and immune diseases are important inducements, and osteocyte necrosis and cystic degeneration in the femoral head are its pathological manifestations. However, the specific mechanism is not yet clear, so there is a series of theoretical arguments. In China, due to the limitation of economic level, education level and medical conditions, the consequences of NONFH (The results of bone biopsy are consistent with ischemic necrosis, but other examinations are normal) are disastrous. Most patients have entered the collapse period and peri collapse period when they go to hospital. Although most scholars have done a lot of research in the field of hip protection and achieved some results, they often miss opportunities. Most patients need to undergo hip replacement surgery, and the economic burden and complications brought by the surgery are too much for patients. In order to achieve early diagnosis and treatment, many researchers have done a lot of work in the field of molecular biology. Through basic and experimental research, the aim is to find relevant molecular biological markers for early detection, diagnosis and effective intervention $Q^{N+1}$ . However, they are all in the bottleneck period and there is no important breakthrough. However, due to the limitations of the research methods of traditional Chinese medicine and the difficulties $K^{N}$ in integrating it with modern molecular biology, the scientific basis provided by most of the studies is not convincing (Calori et al., 2017). The incidence of infection after replacement of femoral head necrosis was 2.1%–10.3%. Because of the huge number of artificial joint replacement operations carried out every year, the number of infected cases is also countless. The infection caused by any reason will have serious consequences, eventually leading to prosthesis loosening and surgical failure. Severe infection can cause paralysis.

$$Q^{N+1}=f\left(Y,M^{N}\right)$$ (1)

$$K^{N}=T+V$$ (2)

$$x_{M}=N^{-1}\left(M_{1}\right)\left(\tau -Y-d\right)$$ (3)

The relative expression level of PI3K/AKT signal pathway is positively correlated with total cholesterol (it is a major steroid compound in mammals and plays an important role in basic cell life activities), low density lipoprotein and other indicators. The more obese patients are, the higher the relative expression level of PI3K/AKT signal pathway is. Clinical research shows that hyperlipidemia and lipid metabolism disorder are one of the high risk factors of femoral head necrosis. Other studies also found that after the application of PI3K inhibitor, the expression of SREBP (Steel Regulatory Element Binding Protein) decreases significantly, which leads to a significant decrease in thyroglobulin indicators. It leads to lipid metabolism disorder and excessive obesity, and even leads to the formation of atherosclerosis (the symptoms of atherosclerosis mainly depend on vascular disease and the degree of ischemia of the affected organs), thus affecting the pathogenesis of femoral head necrosis J (Peng et al., 2021).

$$J=\overset{n}{\underset{i=0}{\sum }}J_{i}$$ (4)

$$N=KS-\frac{CS}{\left\|S+C\right\|}$$ (5)

$$bg=vgS+\frac{k}{\left\|l+m\right\|}$$ (6)

**FIGURE 1:**
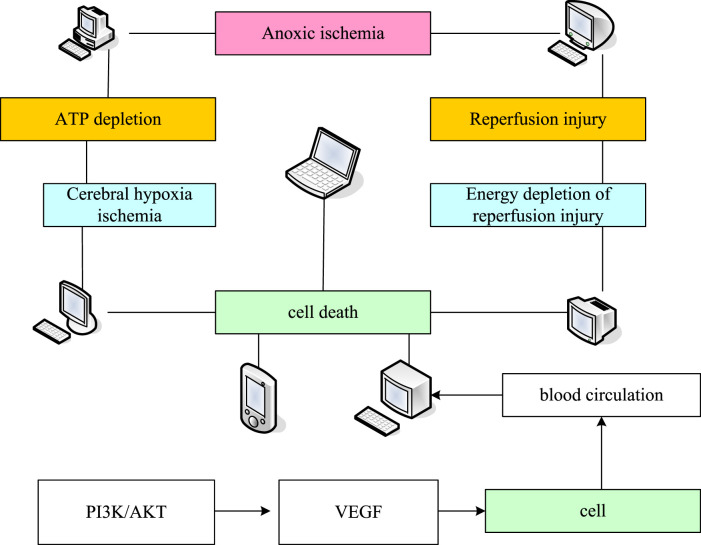
Pathogenesis of NONFH.

AKT combines with eNOS (Endothalial Nitric Oxide Synthase) phosphorylation to promote the production of NO (nitrous oxide). Its upstream factor PTEN (photosynthesis and tensin homolog deleted on chromosomal ten) protein can improve the vascular permeability by affecting the cell growth cycle, so as to adjust the blood circulation around the necrosis of femoral head, thus achieving the role of preventing and treating NONFH. The improvement of vascular permeability (The contraction of endothelial cells is called recurrent transient response because the half-life of the inflammatory mediator of endothelial cell contraction is short and the endothelial cell contraction caused by it is reversible) around the femoral head can provide a certain direction for the treatment of early and middle femoral head necrosis. However, most of them stay in the animal experiment stage, and further research is needed to obtain more intuitive clinical data. The pathogenesis of NONFH is shown in Figure 1.

Studies have shown that PI3K/AKT can regulate the phosphorylation of subunits when activated by external stimulation, thus starting VEGF gene transcription to promote angiogenesis. Moreover, by activating and supplementing VEGF (The gene structure of VEGF family proteins is complex, among which VEGF-A protein is the most studied. The precursor mRNA of VEGF-A gene transcription can form different fragments of VEGF-A protein through alternative splicing), it can promote the high expression of PI3K/AKT, stimulate the production of more VEGF and miRNA protein expression related to angiogenesis in the body, so as to better promote angiogenesis.

PI3K/AKT can directly induce the generation and expression of VEGF. VEGF can promote cell generation and regulate peripheral blood supply, which can help vascular repair and regeneration, and can nourish peripheral bone cells to alleviate the early development of femoral head necrosis. It is possible for VEGF to improve the blood circulation around necrosis of femoral head. The theory is relatively perfect. However, it has not been further applied to clinical treatment. It is worth further discussion and research to better clarify its mechanism of action (Wang et al., 2017; Wen et al., 2020).

The diagnostic criteria is that when the patient meets one of the following three points, the femoral head necrosis can be diagnosed, regardless of whether there are clinical symptoms or signs.

X-ray film: necrotic focus surrounded by sclerotic band and segmental collapse in the femoral head can be observed; Crescent sign and other specific manifestations.

Magnetic resonance imaging (MRI) examination:

T1W1 band low signal, T2W “double line” sign, the lateral low signal band is hyperplastic sclerotic bone, and the medial high signal band is granulation fiber tissue repair.

CT: necrotic focus with clear contour; Subchondral fracture.

### 2.2 MicroRNA-206 induces anoxic necrosis of femoral head

#### 2.2.1 Clinical bone tissue sample

The clinical bone tissue samples of the experimental group used in this paper are from 15 patients with steroid induced avascular necrosis of the femoral head, including nine male patients and six female patients. Another 15 femoral head tissue samples from patients undergoing total hip replacement (The artificial prosthesis, including the femur and acetabulum, is fixed on the normal bone by bone cement and screws to replace the diseased joint) due to femoral neck fracture are used as the control group in this article, including eight male and seven female patients with femoral neck fracture. All patients participating in the study signs the informed consent form, and obtains detailed clinical pathology and prognosis data of all patients in the experimental group and the control group. The study covering relevant patient screening, clinical examination, clinical data acquisition and subsequent experimental analysis. The collected femoral head bone tissue samples are frozen in the prepared liquid nitrogen immediately after the operation for subsequent experimental analysis of total RNA and protein sample extraction.

Cell line: The product classification numbers of hFOB1.19 and human renal epithelial cell line 293 T are CRL-11372 and CRL-3216 respectively.

Main reagents and antibodies: conventional chemical reagents: disodium hydrogen phosphate, methanol, anhydrous ethanol, glacial acetic acid, isopropanol and other conventional reagents used in this paper are analytical pure. Total RNA extraction reagent (Trizol solution) is purchased from Bioengineering Co., Ltd. (Shanghai), and the product number is B511311. Diethyl pyrocarbonate is purchased from Sigma Aldrich (China) Co., Ltd. (Shanghai, China), and the product number is 472565. Agarose is purchased from Yisheng Biotechnology Co., Ltd. (Shanghai, China), and the product number is 10208ES60.

**FIGURE 2:**
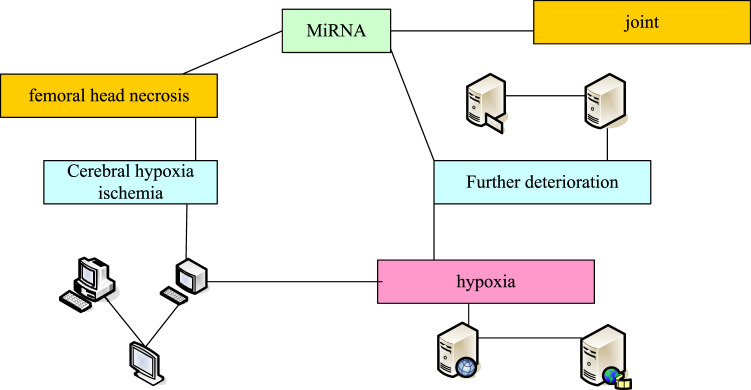
Basic idea of clinical experiment.

DNA marker is purchased from Beyotime Biotechnology Company (Nantong, China), and the product number is D0107. Nucleotide fluorescent dye (SYBR GreenI) is purchased from Thermo Fisher Scientific (China) Co., Ltd. (Shanghai, China), and the product number is S7563. Taq DNA polymerase is purchased from Fullgold Biotechnology Co., Ltd. (Beijing, China), and the product number is AP101-12. The basic idea of clinical experiment is shown in Figure 2.

#### 2.2.2 Total RNA extraction process of bone tissue and cell line

In this paper, all patients with hypoxic avascular necrosis of the femoral head and the contrast group are used to extract total RNA from bone tissue, human osteoblasts and renal epithelial cells using a standardized process. In order to prevent sample contamination and affect the accuracy of experimental results, consumables such as gun heads and centrifuge tubes used in the extraction process are soaked in DEPC water (which is used for dissolution of RNA precipitation and various reaction systems containing RNA, such as reverse transcription, annealing of siRNA, etc.) in advance. The specific extraction process is as follows:

(1) Tissue and cell homogenate treatment: For bone tissue samples, the bone tissue samples stored in liquid nitrogen tank shall be ground in liquid nitrogen immediately after being taken out until uniform fine particles are formed. Then, pre cooled TRIzol (which is an RNA extract that can be directly extracted from cells to RNA) solution (1 mL of TRIzol solution is added to every 0.1 g of bone tissue) is added to fully mix, so that RNA samples can be fully dissolved in TRIzol solution. For cultured human osteoblasts and renal epithelial cells, 800 g of them are centrifuged for 5 min to collect cells, and then an appropriate amount of pre cooled PBS solution is added to resuspension cells. PBS solution is removed by centrifugation, and cells are washed twice in this way. Finally, about 1 mL TRIzol solution is added to resuspension cells and repeated blowing to promote cell lysis. The volume of reagent and solution used in the following experimental process is calculated according to 1 mL of homogenate.
(2) The centrifuge tube is inverted onto a clean absorbent paper, and then it is left to dry at room temperature. Finally, about 40 ul double distilled water treated by DEPC is added, and the sample is shaken on a vortex oscillator for a short time to promote the dissolution of the sample. It is stored at −80°C for standby.
(3) Agarose gel electrophoresis: 2 μL of the obtained RNA sample is taken out and separated by 2% agarose gel electrophoresis and photographed as v . The quality a and integrity of the obtained sample dP are analyzed (Zhu et al., 2020).

$$a=\sin\frac{2mt}{c}$$ (7)

$$v=\frac{at}{2\pi }\cos\frac{2vt}{t}$$ (8)

$$dP={dq}_{x}+{dq}_{y}$$ (9)

(4) Determination of RNA sample solubility: Micropipette is used to suck 1 μL extracted RNA sample, and measure the RNA sample solubility and A260/A280 ratio through micro spectrophotometer. It is RNA sample lp meeting the purity requirements (Zhang M. et al., 2017; Yan et al., 2018).

$$K=d+y_{m}$$ (10)

$$o_{total}=\frac{o_{F}}{c}$$ (11)

$$lp=\frac{k_{VS}}{k_{\max}}=\frac{v_{M}}{b_{VS}}$$ (12)

#### 2.2.3 Extraction of total protein from bone tissues and cell lines

In order to analyze the changes of protein levels of related genes, the animal whole protein extraction kit (product number: C510003) produced by Shanghai Sangong Biotechnology Co., Ltd. is used to extract the total protein of femoral head tissue, human osteoblasts and renal epithelial cells. The specific extraction process is described below. Extraction process of total protein from femoral head is:

(1) Reagent preparation: Lysis buffer provided in the kit is stored at 4°C. Protease inhibitor, phosphatase inhibitor and benzylsulfonyl fluoride solution are stored at—20°C. Before protein extraction, the relevant reagents are placed on ice for several minutes.
(2) In the precooled Lysis Buffer, phosphatase inhibitor (5 μL/mL), protease inhibitor (5 μL/mL) and benzylsulfonyl fluoride solution (10 μL/mL) are added, fully mixed and evenly placed on ice for several minutes for standby.
(3) The femoral head tissue stored in liquid nitrogen tank is taken out. In liquid nitrogen, all bone tissue blocks are broken and ground into fine powder. After the liquid nitrogen volatilizes completely, the precooled Lysis buffer is added to continue grinding for several minutes to form tissue homogenate.
(4) The tissue homogenate obtained is transferred to a precooled 1.5 mL Eppendorf centrifuge tube, left on ice for 15 min. It is subjected to severe eddy vibration for 3–5 times to fully extract the protein.
(5) After centrifugation at 4°C for 10 min, the supernatant is transferred to a new precooled 1.5 mL Eppendorf centrifuge tube. The obtained sample is the total protein of the tissue. After the protein solubility is determined, it is packaged and stored at—70°C for standby.

Necrosis of the femoral head can cause the increase of C-reactive protein, suggesting that infection: the important clinical significance of the increase of C-reactive protein is to prompt infection, especially bacterial infection, which will lead to a significant increase of C-reactive protein. Virus infection usually has a slight increase or normal level of C-reactive protein, so C-reactive protein can be used as a differential indicator of infection.

#### 2.2.4 HFOB1.19 osteoblast transfection method

First, the cells inoculated with hFOB1. 19 cells are placed on a 24 well cell culture plate. The cells with a confluence of about 50%–60% are washed twice with PBS solution, and 300 μL serum free medium is added to each cell culture hole to continue to culture (5% CO_2_, 37°C). The basic culture medium without serum is diluted with Lipofectamine 2000 reagent at a ratio of 50:1 (to ensure the demand of each cell culture hole 50 μL), and diluted with the basic culture medium without serum. The target DNA fragments to be transfected are diluted at an appropriate ratio (to ensure each cell culture hole 50 μL), and left at room temperature for several minutes. Subsequently, the two diluted solutions are mixed by the same volume and left at room temperature for 20 min. Finally, each cell culture well is dripped with the mixed dilute solution obtained from the above 100 μL and gently mixed. It is continuously cultured for about 6 h and replaced with fresh medium for further culture. Cells are collected for subsequent cytological and molecular biological correlation analysis for about 24 h.

#### 2.2.5 Cell activity test method (CCK8 method)

Cell Counting Kit-8 (CCK8) method is used to detect the cell viability of cultured human hFOB1. 19 osteoblasts. The experimental operation process is carried out according to the technical methods provided in the manual. The simple experimental process is as follows. After transfection, hFOBl. 19 osteoblasts are cultured for 24–27 h and used for CCK-8 analysis of cell activity. Cells in each group are collected at three different time points, namely, 24, 48 and 72 h. First, pipettes are used to gently remove old media. Then, according to the ratio of 100:1, the fresh complete culture medium is diluted with CCK-8 reagent. Finally, the cell culture plate $n\left(j\right)$ is put back into the incubator to continue to culture for about 1 h, and then detected by the microplate reader for statistical analysis of the difference $c_{y}$ in cell activity among groups. At least three biological repeats shall be set for the above analysis (Wang et al., 2019).

$$k_{g}=g_{FS}-g$$ (13)

$$n\left(j\right)=\frac{∆l}{∆z*n}$$ (14)

$$a_{as}=\frac{1}{d\left(\lambda \right)}$$ (15)

$$c_{y}=h+\frac{g}{P_{\max}∙t_{0}}$$ (16)

During the miR-206 expression level difference experiment, the femoral head tissue samples of 15 patients with femoral neck fracture who underwent total hip arthroplasty were collected as the control group.

### 2.3 Statistical treatment

Pearson correlation method (which can measure the correlation between vectors) is used to analyze the correlation between the parameters between groups. In this paper, *p* < 0.05 and *p* < 0.01 are used as the criteria for evaluating statistically significant and extremely significant differences (Wu et al., 2018; Petek et al., 2019).

$$q_{D}=\frac{2\varepsilon_{s}}{j*h}\sqrt{\frac{m_{A}m_{D}}{m_{A}{+m}_{D}}}$$ (17)

$$f_{bi}=\ln\frac{kbj_{D}}{m_{i}}$$ (18)

$$\eta =\frac{v}{ls/m_{0}}$$ (19)

$$d=\overset{n}{\underset{i=0}{\sum }}\left(g_{i}\omega_{i}+c_{i}\right)$$ (20)

## 3 Results of microRNA-206 induced hypoxic necrosis of femoral head

In this paper, the reverse transcription of mRNA is carried out using EasyScripte reverse transcriptase produced by Beijing Quanshijin Biotechnology Co., Ltd. The extracted RNA sample 1 μg is used as the template, and the reaction system is 7 μL. The prepared reaction system is fully mixed evenly. Ordinary PCR (Polymerase Chain Reaction, which can amplify DNA, making a small amount of DNA become more) instrument is used for cDNA synthesis. The specific reaction procedure is: 70°C, 4 min; 37°C, 55 min; 95°C for 4 min. The synthesized cDNA samples can be used for quantitative PCR analysis or stored at—15°C for future use. The reverse transcription system is shown in Table 1.

**TABLE 1:** Reverse transcription system.

Reaction system	Unit (μL)
RNA samples	1
OligodT primers (18)	1
dNTP mixed solution	0.5
Reverse transcriptase	0.5
DEPC water	2
Reverse transcription buffer	2

**FIGURE 3:**
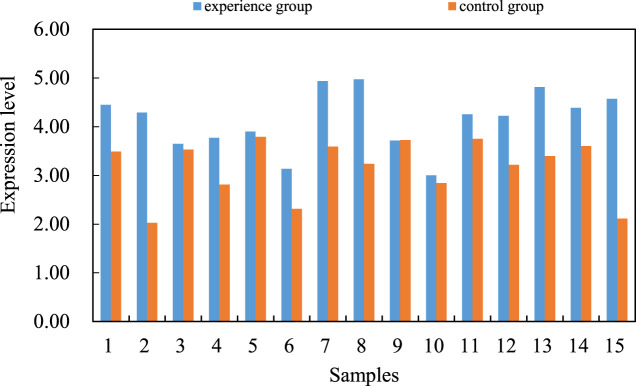
Differences in miR-206 expression levels.

At the same time, the femoral head tissue samples of 15 patients with femoral neck fracture undergoing total hip replacement are collected as the control group. Total RNA samples from femoral head tissues of two groups of patients are extracted. The total cDNA samples of each sample are obtained by reverse transcription. The expression level of miR-206 between the experimental group and the control group is compared by real-time fluorescent quantitative PCR. The difference of miR-206 expression level is shown in Figure 3. The expression level of the experimental group was 4.1, and that of the control group was 3.2.

**FIGURE 4:**
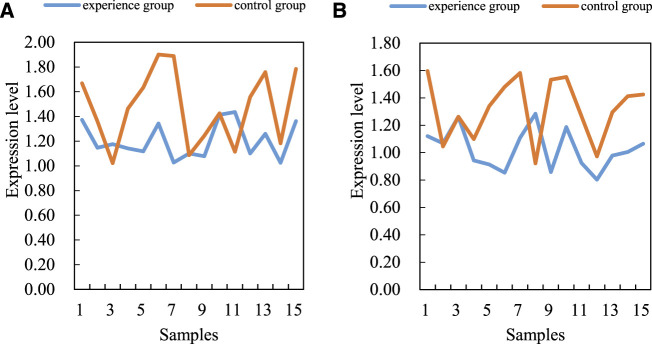
mRNA and protein levels of VEGF/PI3K/AKT signaling pathway. **(A)** MRNA **(B)** Protein.

The difference of VEGF/PI3K/AKT signal pathway gene expression level between the two groups of bone tissues by fluorescence quantitative PCR and western blotting is compared (the mRNA level of VEGF/PI3K/AKT signal pathway is shown in Figure 4A. The average data of the experimental group is 1.2, and the average data of the control group is 1.5. The regulation of this gene expression may be an important molecular pathological mechanism for the occurrence and development of steroid induced avascular necrosis of the femoral head (the level of VEGF/PI3K/AKT signal pathway protein is shown in Figure 4B). The mRNA and protein levels of VEGF/PI3K/AKT signaling pathway are shown in Figure 4.

**FIGURE 5:**
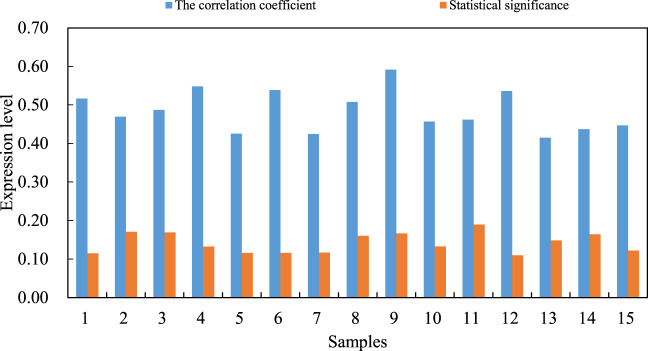
Interaction between miR-206 and VEGF/PI3K/AKT signal pathway genes.

Results are analyzed by real-time fluorescent quantitative PCR and western blotting. In order to verify this, SPSS software (SPSS is the first statistical software in the world to use graphical menu driven interface. Its most prominent feature is that the operation interface is extremely friendly and the output results are beautiful) is used to analyze the correlation between miR-206 and VEGF/PI3K/AKT signal pathway mRNA levels in steroid induced avascular necrosis of the femoral head. There is a significant negative correlation between miR-206 and VEGF/PI3K/AKT signal pathway mRNA levels in femoral tissue of steroid induced avascular necrosis of the femoral head. The correlation coefficient (R value) is about 0.48, which is statistically significant. There may be some interaction between miR-206 and VEGF/PI3K/AKT signaling pathway genes. The interaction between miR-206 and VEGF/PI3K/AKT signal pathway genes is shown in Figure 5.

**FIGURE 6:**
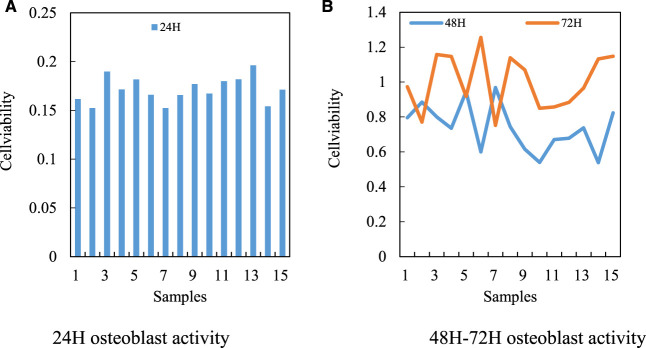
Regulation of miR-206 expression level on osteoblast activity. **(A)** 24H osteoblast activity **(B)** 48H–72H osteoblast activity.

Based on the previous research on miR-206, and the specific binding ability of miR-206 and VEGF/PI3K/AKT signal pathway genes to start specific regions, two kinds of osteoblasts with different miR-206 expression levels are constructed in human hFOB1. 19 osteoblasts through the Lipofectamine TM2000 transfection system to study the regulatory role of miR-206 in the cellular processes of osteoblast activity, proliferation and apoptosis in detail. At the same time, the downstream regulatory mechanism of miR-206 regulating osteoblast biological function is studied with the above cell materials, especially the possible role of VEGF/PI3K/AKT signaling pathway in the regulation of osteoblast function by miR-206. First, the effect of miR-206 expression level on hFOB1. 19 osteoblast activity is analyzed by Cell Counting Kit-8 (CCK8) experiment. After human hFOB1. 19 osteoblasts are transfected with miR-206 mimics, miR-206 inhibitor and control fragments, the cell viability of hFOB1.19 osteoblasts is detected by CCK-8 at 24, 48 and 72 h, respectively. The expression level of miR-206 in human osteoblasts is negatively correlated with its cell activity. It shows that miR-206 has the effect of inhibiting osteoblast activity (the activity of 24 H osteoblast is shown in Figure 6A, 48H–72H osteoblast activity is shown in Figure 6B. The regulation of miR-206 expression level on osteoblast activity is shown in Figure 6.

**FIGURE 7:**
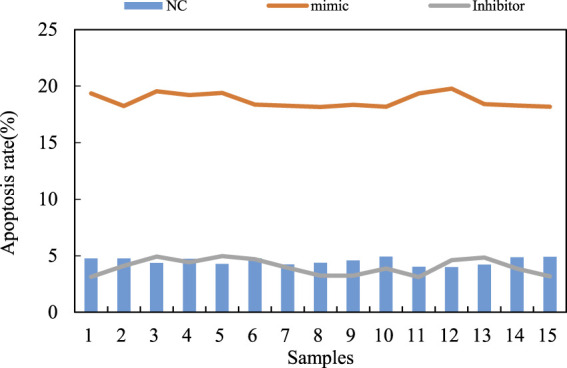
Regulation of miR-206 expression level on osteoblast apoptosis.

The dynamic balance between osteoblasts and osteoclasts is the main mechanism to maintain the normal function of human bones. In order to further understand the cytological mode of miR-206 involved in the pathological process of steroid induced avascular necrosis of the femoral head, the apoptosis of human hFOB1. 19 osteoblasts overexpressed and silenced miR-206 are further analyzed. First, the apoptosis of osteoblasts transfected with MicroRNA-206 mimics and MicroRNA-206 inhibitor is compared and analyzed by Hoechst 33258 staining method. In particular, miR-206 mimics transfected human hFOB1. 19 osteoblasts leads to extremely significant apoptosis. Apoptosis is hardly observed in osteoblasts of miR-206 inhibitor transfection group and normal control group. This result shows that overexpression of miR-206 can promote apoptosis of human hFOB1. 19 osteoblasts. At the same time, the apoptosis of the three groups of human osteoblasts is detected by flow cytometry. After Annexin V and propidium iodide staining, flow cytometry is used to quantitatively analyze the proportion of apoptotic cells in the three groups. The expression level of MicroRNA-206 has a strong promoting effect on osteoblast apoptosis. The regulatory effect of miR-206 expression level on osteoblast apoptosis is shown in Figure 7.

**FIGURE 8:**
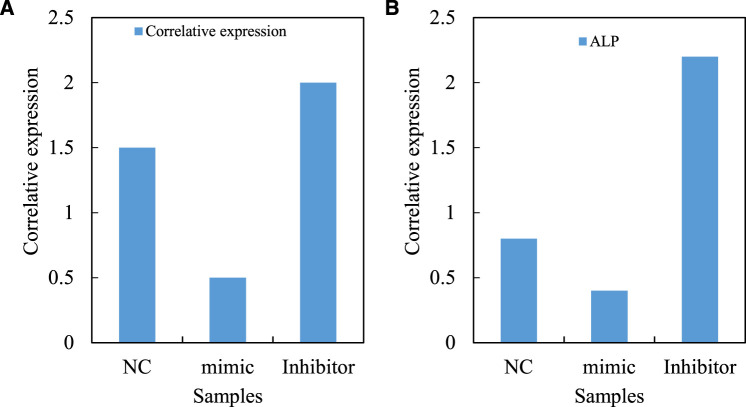
Regulation of hypoxic avascular necrosis of femoral head. **(A)** VEGF/PI3K/AKT signal pathway **(B)** ALP.

The expression level of VEGF/PI3K/AKT signal pathway gene in osteoblasts transfected with MicroRNA-206 mimics and MicroRNA-206 inhibitor is first analyzed (VEGF/PI3K/AKT signal pathway is shown in Figure 8A). Quantitative RTPCR (reverse transcription polymerase chain reaction) analysis shows that the mRNA level of VEGF/PI3K/AKT signal pathway gene in osteoblasts transfected with MicroRNA-206 mimics is significantly downregulated. On the contrary, the mRNA level of VEGF/PI3K/AKT signal pathway gene in osteoblasts transfected with MicroRNA-206 inhibitor increases dramatically. These results indicate that MicroRNA-206 has a strong negative regulatory effect on the mRNA level of VEGF/PI3K/AKT signaling pathway genes. Alkaline phosphatase (ALP) is a major marker gene for osteogenic differentiation of bone marrow mesenchymal stem cells. The study also finds that the expression level of this gene in osteoblasts is also significantly regulated by MicroRNA-206, and MicroRNA-206 mimics significantly inhibits the expression of ALP gene. MicroRNA-206 inhibitor significantly promotes the expression level of ALP gene (ALP is shown in Figure 8B). The regulation of hypoxic avascular necrosis of the femoral head is shown in Figure 8.

ALP is marked with green fluorescence. The osteoblasts are intervened with 10–6 mol/L MicroRNA 206 for 0, 12, 24 and 48 h, and then immunofluorescence staining is performed. The expression of ALP fluorescence decreases significantly at 12, 24 and 48 h. There is only a small amount of immunofluorescence expression in 0 h group. The fluorescence intensity of caspase-3 is higher in the treatment group treated with MicroRNA 206 for 24 and 48 h than that in the control group (*p* < 0.05). MicroRNA-206 can upregulate caspase-3 expression and induce osteoblast apoptosis in a time dependent manner. The absorbance contrast is shown in Table 2.

**TABLE 2:** Comparison of absorbance.

Time (h)	ALP (mol/L)	*p*
48	8	<0.05
24	11	
12	15	
0	20	

Then, in the logarithmic growth period, the necrosis cells of the femoral head are taken after hypoxia in good growth condition. The cell density to 2X 104/well is adjusted with the third time medium, and is connected to the 96 well plate. Then the MTT detection method is used. The medium is sucked out and 150 ul DMSO (Dimethyl sulfide) is added to shake for 10 min. The absorbance value OD568 of each hole is measured by microplate reader. Compared with miR-206 + VEGF/PI3K/AKT group, the average cell proliferation rate of VEGF/PI3K/AKT pretreatment group increases significantly (*p* < 0.05). Compared with the control group, the average cell increment rate of the pretreatment group has no significant change. The relative increment rate of cells under different experimental conditions is shown in Table 3.

**TABLE 3:** Relative cell growth rate under different experimental conditions.

Group	For the first time	The second time	Third time
Blank	0.07	0.069	0.068
Control	1.4	1.3	1.4
miR-206	1.1	1.2	1.1
VEGF⁄PI3K⁄AKT	1.6	1.5	1.7
miR-206 + VEGF⁄PI3K⁄AKT	1.3	1.3	1.2

The chemotherapy drug delivery system has laid the foundation for personalized therapy. In view of the high accuracy of this method, it can determine the optimal dose of drugs for patients, thereby reducing side effects and reducing treatment costs. This paper also considers the targeted drug delivery of the chemotherapy drug delivery system. The first antibody is diluted with sealing solution, so that PVDF (polyvinylidene fluoride) film is immersed in the first antibody incubation solution, and incubated overnight in a 4°C incubator. The dilution concentration of antibody is shown in Table 4.

**TABLE 4:** Diluted concentration of antibody.

Name	Dilution ratio
LC3	1:1,000
Beclin1	1:1,000
P62	1:1,000
AKT	1:1,000
P-AKT	1:1,000

**FIGURE 9:**
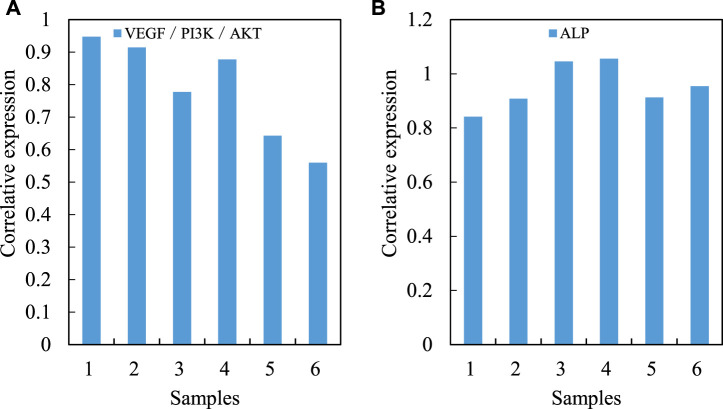
Comparison of regulatory effects of MicroRNA-206. **(A)** VEGF/PI3K/AKT signal expression **(B)** MicroRNA-206 inhibitor transfection of ALP gene in osteoblasts.

In the process of regulating the apoptosis, proliferation and differentiation of human osteoblasts by MicroRNA 206, VEGF/PI3K/AKT signaling pathway and other important regulatory factors related to apoptosis and differentiation have undergone significant changes. A specific siRNA (Small interfering RNA) targeting VEGF/PI3K/AKT signaling pathway gene is designed. At the same time, based on the previous research results about the significant increase of gene expression level of VEGF/PI3K/AKT signal pathway in human osteoblasts transfected with MicroRNA 206 inhibitor, MicroRNA 206 inhibitor and VEGF/PI3K/AKT signal pathway are co transfected into human hFOB1. 19 osteoblasts, with a view to building a human osteoblast strain co silenced with MicroRNA 206 and VEGF/PI3K/AKT signal pathway genes. It is used to compare with the osteoblasts transfected with MicroRNA 206 inhibitor, and to evaluate the regulatory role of VEGF/PI3K/AKT signaling pathway in the process of apoptosis of osteoblasts mediated by MicroRNA 206 and its molecular mechanism. Quantitative RT-PCR analysis shows that the mRNA level of VEGF/PI3K/AKT signal pathway gene in hFOB1.19 osteoblasts co transfected with MicroRNA 206 inhibitor and VEGF/PI3K/AKT signal pathway decreases significantly compared with human osteoblasts transfected with MicroRNA 206 inhibitor alone. It indicates that this cell line can be used for subsequent research. At the same time, quantitative RT-PCR analysis confirms the expression of ALP gene in osteoblasts transfected with MicroRNA-206 inhibitor. (VEGF/PI3K/AKT signal expression is shown in Figure 9A. The ALP gene in the osteoblasts transfected by MicroRNA-206 inhibitor is shown in Figure 9B. The comparison of regulatory effects of MicroRNA-206 is shown in Figure 9.

**FIGURE 10:**
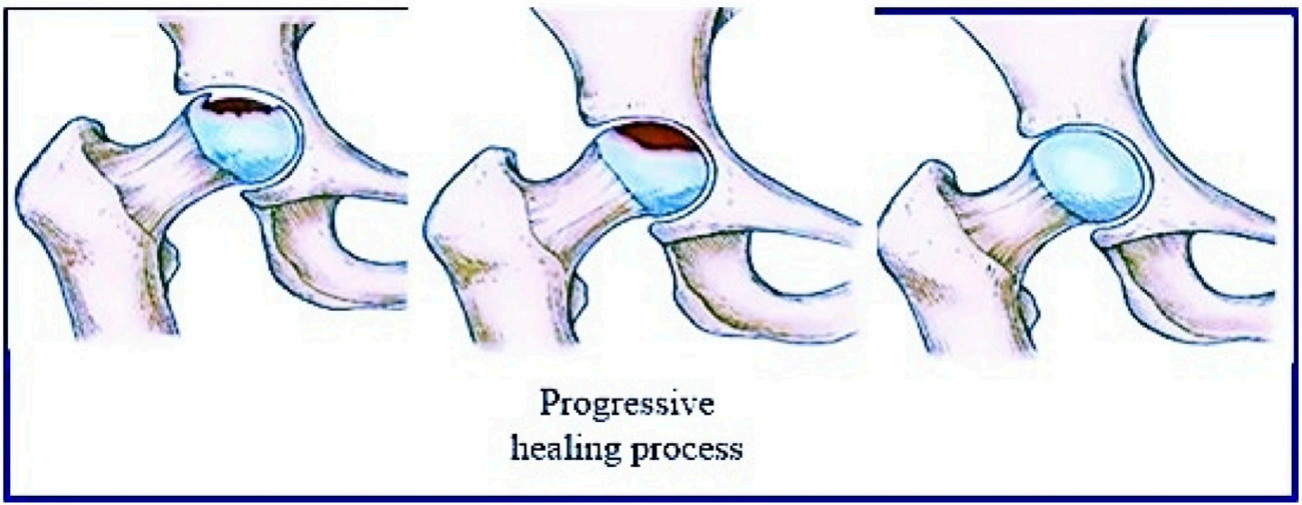
The femoral head gradually healed after necrosis (https://xn7p.cn/0f75e).

The osteoblasts are intervened with 10–8 mol/L, 10–7 mol/L, 10–6 mol/L MicroRNA 206 for 12, 24 and 48 h, and then CCK8 is detected. The results shows that after 12 h of intervention, 10–7 mol/L, 10–6 mol/L MicroRNA-206 can significantly reduce the cell proliferation activity. 10 * mol/LMicroRNA-206 has no significant inhibitory effect on osteoblast proliferation (*p* > 0.05). Compared with 10–8 mol/L group, the proliferation activity of 10–7 mol/L and 10–6 mol/L MicroRNA 206 groups is significantly decreased (*p* < 0.05). Compared with 10–7 mol/L group, the proliferation activity of 10–6 mol/L MicroRNA-206 osteoblasts decreases significantly (*p* < 0.05). It is suggested that MicroRNA 206 inhibits osteoblast proliferation in a dose-dependent manner. At the same concentration of MicroRNA 206, the relative proliferation rate of osteoblasts decreases gradually with the prolongation of the time of MicroRNA 206 intervention for 12, 24 and 48 h. The results shows that MicroRNA 206 inhibits the proliferation of osteoblasts. The longer the action time is, the more obvious the inhibition is. The influence difference of different time and concentration is shown in Table 5. The femoral head gradually healed after necrosis, as shown in Figure 10.

**TABLE 5:** Difference of influence of different time and concentration.

Group	12 h	24 h	48 h
10^−6 ^mol/L	0.46	0.61	0.76
10^−7 ^mol/L	0.48	0.62	0.79
10^−8 ^mol/L	0.49	0.72	0.99
Contrast	0.51	0.71	1.12

## 4 Conclusion

This article has explained the correlation between constitution and NONFH at the molecular level, which is conducive to the play of the unique advantages of the theory of “treating diseases before occuring” in exploring the prevention and revealing the causes of NONFH. It is the direction of continuous development of medicine to explore the mechanism of NONFH blood circulation disorder from the perspective of molecular biology, understand the causes of the disease from a microscopic perspective, and obtain treatment methods. PI3K/AKT can delay and prevent femoral head necrosis by correcting abnormal lipid metabolism, enhancing vascular permeability and promoting vascular regeneration. In this paper, the research on PI3K/AKT promoting vascular regeneration by enhancing VEGF was relatively more complete, and the relevant evidence was also more sufficient. This paper fully discussed the safety and feasibility of the chemotherapy drug delivery system through local tissue absorption of chemotherapy drugs. However, it is still in the laboratory research stage, and the effect of PI3K/AKT on NONFH bone tissue needs to be improved. At the same time, obesity, genetics and environmental factors can also be studied from a microscopic perspective through signal pathways, so as to more clearly understand the mechanism and treatment of NONFH. It is the future direction to know and understand the pathological changes and pathogenesis of NONFH more accurately to help prevent and treat it in advance.

## Data Availability

The original contributions presented in the study are included in the article/supplementary material, further inquiries can be directed to the corresponding author.
